# News and Events of the Week

**Published:** 1911-03-18

**Authors:** 


### NEWS AND EVENTS OF THE WEEK.

THE Battersea Borough Council has decided to destroy the Brown Dog Memorial and to retain the drinking fountain after erasing the inscription.

AT the annual dinner of the Medical Society of London last week Mr. F. W. Mott, F.R.S., M.B., B.S., was presented with the Fothergillian Gold Medal for his work on "The Affections of the Nervous System."

THE Museum of the Royal College of Surgeons is to be added to by the reconstruction of a house adjoining it. The new rooms will contain the proposed Medico-Legal Collection and other specimens which will be displaced by the rearrangement and enlargement of the Pathological Collection.

DURING the past year thirty-one women students of medicine were admitted to the ranks of the doctors on the roll of the Faculty of Medicine of Paris. Of these, eleven were of French nationality, the remainder being foreigners.

THE French Government has taken the initiative in arranging an international sanitary conference to be held in Paris in May. Points for discussion are being arranged by the Committee of the Office International de l'Hygiène Publique, of which Dr. Poltevin is Director and Dr. Santoliquido the President.

SPECIMENS of standard bread are on view in the Food Section of the Institute of Hygiene, 34 Devonshire Street, Harley Street, W. Visitors to the exhibition, which is free to the public, can have the properties, composition, and chief characteristics of good standard bread explained to them by the medical superintendent.

THE collision in Portland harbour on July 6 last between the auxiliary hospital ship *Maine* and Mrs. Jones' schooner *Gordon* has been made the subject of an action. In the Admiralty Division, before Mr. Justice Bargrave Deane, last Saturday, Mrs. Jones sought against Commander A. C. Dunne to recover the amount of the damage done to the *Gordon* in the collision. His Lordship, though with some doubt, held that the *Maine* was alone to blame.

STEPS have been taken to celebrate the scientific jubilee of Professor Armand Gautier by striking a commemorative medal. The Professor is a member of the Institute and of the Academy of Medicine, as well as professor at the Paris Faculty of Medicine and president of the Academy of Sciences. He has now been teaching for fifty years at the University, and has devoted the whole of his life to science. A committee has been formed to put the project into execution. All subscribers of £1 will receive a bronze replica of the jubilee medal. The treasurer of the committee is M. P. Masson, 120 Boulevard Saint-Germain, Paris.

THE stream of new medical journals in France would seem to be unceasing. The first number of *Æsculape*, a monthly illustrated review (which proposes to take an interest not only in purely medical matters, but also in subjects borrowed from the sciences and arts, provided that they exhibit what may be called paramedical, or rather "pariatrical" qualities), has just appeared, under the editorship of M. Rouzaud, editor of the "Index Medicus." A less ambitious organ, yet one which promises to be of local interest, is *La Tunisie Médicale*. A third, *L'Echo Médical et Pharmaceutique*, a monthly periodical, which is edited from 324 rue Saint-Martin, Paris, intends to devote its energies to reviewing scientific and professional questions in the domain of pharmacy.

DR. T. D. LISTER read a paper before the members of the Society of Medical Officers of Health on Friday last week on "The Treatment of Phthisis by Industrial Insurance, with special reference to the Benenden Sanatorium." The meeting was held at 1 Upper Montague Street, W.C., and Dr. Willoughby presided.

SIR THOS. BARLOW, President of the Royal College of Physicians, speaking at the annual meeting of the New Hospital for Women, said during the past two years women had at length overcome one of the last obstructions in the full accomplishment of their desire to be admitted into the medical profession. With patience, determination, and dignity beyond all praise, they had so worked that at last they had induced the College of Physicians to admit them to the examination of that body. As a humble representative of that College, which he took the liberty of maintaining to be, in many ways, the most important medical body within this Kingdom, he would say that he had come that day to congratulate them on the position they had earned, and the opportunities which they had taken advantage of in regard to recognition in medicine. He thought that with the admission of women to the full privileges of membership and fellowship of the Royal Society of Medicine, nearly all the difficulties in regard to their proper status had been overcome. (Cheers.) It was true that there were still appointments which had not yet been open to medical women, but year by year, week by week, those difficulties were being surmounted. Women were being appointed to many of the medical offices, and there were openings up in the Medical Inspectorship of Schools for them, and many other appointments he could mention which make an enormous alteration in the opportunities for useful work for their side of the profession. (Cheers.)

A SPECIAL clinical demonstration of interesting cases will be given by Dr. Percy Jakins at the Central London Throat and Ear Hospital, Gray's Inn Road, W.C., at 3.30 P.M. on Friday, March 24.

THE following are the arrangements for next week at the Medical Graduates' College and Polyclinic, 22 Chenies Street, W.C.: Monday, March 20, at 4 P.M., Mr. J. E. R. MacDonagh, Clinique (Skin); at 5.15 P.M., Dr. J. L. Bunch, "Some Common Skin Diseases in Children and their Treatment." Tuesday, at 4 P.M., Dr. Lewis Smith, Clinique (Med.); at 5.15 P.M., Dr. S. Russell Wells, "Angina Pectoris." Wednesday, at 4 P.M., Mr. G. E. Waugh, Clinique (Surg.); at 5.15 P.M., Dr. G. H. Savage, "Morbid Mental Growths." Thursday, at 4 P.M., Dr. E. Cautley, Clinique (Med.); at 5.15 P.M., Dr. G. E. Herman, "Endometritis." Friday, at 4 P.M., Mr. Sydney R. Scott, Clinique (Ear, Nose, and Throat).

THE following are the arrangements for next week at the West London Postgraduate College, West London Hospital, Hammersmith Road: March 20 and 23, at 10 A.M., Demonstration by Surgical Registrar; March 20, at 12 NOON, Pathological Demonstration; March 21, at 11.30 A.M., Demonstration of Minor Operations; March 22, at 10 A.M., Gynæcological Demonstration; March 23, at 12.15 P.M., Lecture, Dr. Grainger Stewart, Practical Medicine. Lectures at 5 P.M.: March 20, Mr. Bidwell, Minor Surgery; March 21, Mr. Pardoe, "Retention of Urine"; March 22, Mr. Bidwell, Minor Surgery; and March 23, Mr. Bidwell, Minor Surgery.

The Astley Cooper Prize for 1913, being the next triennial prize of £300, under the will of the late Sir Astley P. Cooper, will be awarded to the author of the best essay or treatise on "The Means by which the Coagulability of the Blood may be altered." The competition is open to everyone except the staff of Guy's and St. Thomas's Hospitals and their relations; but the essay must not be the production of two or more authors. Essays, written in English, must be sent to Guy's Hospital addressed to the physicians and surgeons, on or before January 1, 1913. Particulars may be obtained from Sir Cooper Perry, M.D., Guy's Hospital, London.

Last Wednesday at Clerkenwell Ralph Campbell Mackenzie, seventy, described as an ex-Army surgeon, F.R.C.S., F.R.C.P., M.D., was charged with feloniously marrying Sybil Rhoda Duncombe on February 2, 1911, his wife, Ellen Gordon Mackenzie, being then and now alive. The prisoner was originally charged with obtaining 3s. 9½d. by false pretences from Gabrielle Sieffert, a lodging-house keeper in St. Pancras. It was then stated that he described himself as "Major-General Viscount Mackenzie" and the "Duke of St. Omars." Sybil Rhoda Duncombe, of High Road, Chiswick, said she was an artist. On February 2 she went through a form of marriage with him. They were married by special licence. He said he had estates in Scotland, and that he was the "Duke of St. Omars." He made a will in her favour. They lived together till February 28, when he took her to Liverpool, where he was arrested. Mr. d'Eyncourt (to prosecutrix): Did you believe he would leave you £10,000 a year and a castle, and jewels and horses?—Yes, I did for the time. Is he in his right mind?—Oh, yes. The case was remanded.

